# Assessment and Improvement of Avatar-Based Learning System: From Linguistic Structure Alignment to Sentiment-Driven Expressions

**DOI:** 10.3390/s25061921

**Published:** 2025-03-19

**Authors:** Aru Ukenova, Gulmira Bekmanova, Nazar Zaki, Meiram Kikimbayev, Mamyr Altaibek

**Affiliations:** 1 Faculty of Information Technologies, L.N.Gumilyov Eurasian National University, Astana 010000, Kazakhstan; gulmirara@gmail.com (G.B.); mameralt@outlook.com (M.A.); 2 College of Information Technology, United Arab Emirates University (UAEU), Al Ain 15551, United Arab Emirates; nzaki@uaeu.ac.ae; 3 Faculty of Journalism and Social Sciences, L.N.Gumilyov Eurasian National University, Astana 010000, Kazakhstan; kikimbayev.meiram1983@gmail.com

**Keywords:** avatar-based learning system, sentence-based gesture mapping, e-learning, sentiment analysis, emotion

## Abstract

This research investigates the improvement of learning systems that utilize avatars by shifting from elementary language compatibility to emotion-driven interactions. An assessment of various instructional approaches indicated marked differences in overall effectiveness, with the system showing steady but slight improvements and little variation, suggesting it has the potential for consistent use. Analysis through one-way ANOVA identified noteworthy disparities in post-test results across different teaching strategies. However, the pairwise comparisons with Tukey’s HSD did not reveal significant group differences. The group variation and limited sample sizes probably affected statistical strength. Evaluation of effect size demonstrated that the traditional approach had an edge over the avatar-based method, with lessons recorded on video displaying more moderate distinctions. The innovative nature of the system might account for its initial lower effectiveness, as students could need some time to adjust. Participants emphasized the importance of emotional authenticity and cultural adaptation, including incorporating a Kazakh accent, to boost the system’s success. In response, the system was designed with sentiment-driven gestures and facial expressions to improve engagement and personalization. These findings show the potential of emotionally intelligent avatars to encourage more profound learning experiences and the significance of fine-tuning the system for widespread adoption in a modern educational context.

## 1. Introduction

Avatars have emerged as a revolutionary tool in educational technology, providing new opportunities to improve the interactivity and personalization of learning systems. In Kazakh language education, where cultural and linguistic nuances are essential, avatars can offer learners a relevant and supportive atmosphere. These virtual avatars mimic human-like interactions, applying visual signals such as facial expressions and gestures to increase engagement and learning results [1].

Facial expressions and gestures significantly impact digital learning [2], particularly for low-resource languages such as Kazakh. Nonverbal communication strategies bridge emotional and cognitive gaps, allowing students to stay engaged and connected to the topic.

The potential of avatars is enhanced when they dynamically adjust to the linguistic and emotional context of the learner’s input [3]. This is especially important in Kazakh, where sentence structure and emotional tone are intricately linked. By combining linguistic and structural alignment with sentiment-driven expressions, avatars can generate grammatically correct responses while being culturally and emotionally suitable. This article explores the creation of an advanced avatar-based learning system for Kazakh education. It demonstrates how integrating linguistic alignment and sentiment analysis can make learning systems more successful and human-centered, addressing learners’ requirements while building a stronger connection between them and the content.

Creating an avatar-based learning system consistent with linguistic frameworks presents substantial hurdles, especially for low-resource languages like Kazakh. Kazakh’s grammatical complexity and distinct syntactic patterns necessitate exact language alignment to enable genuine and relatable interactions. The initial experiments focused on aligning avatar behaviors with these language patterns to improve sentence clarity and engagement. However, while this method produced grammatically correct responses, it lacked emotional depth and adaptability, limiting the avatar’s potential to connect with learners empathetically.

Kazakhstan lacks the robust sentiment analysis techniques and datasets which are required for building emotionally expressive avatars. Sentiment analysis is critical in determining the emotional tone of a learner’s input, allowing avatars to adjust their expressions and gestures dynamically. Without such resources, avatars are limited to predefined, basic behaviors that do not convey the subtle interaction of verbal meaning and emotional tone. This limitation has an impact not only on avatar expressiveness but also on their ability to inspire learner engagement and motivation. To remedy this, sentiment analysis should be integrated into the avatar-based system, allowing for context-aware and emotionally resonant interactions suited to the Kazakh language’s linguistic and cultural specifics.

This article focuses on improving the avatar-based learning system by transitioning from basic language alignment to more complex, sentiment-driven expressions. Drawing on recent advances in sentiment analysis for Kazakh texts, the proposed approach combines a semantic knowledge base and a hybrid sentiment analysis model to generate avatars that can dynamically adjust their behaviors based on both the input’s linguistic structure and emotional tone. This approach attempts to make avatars grammatically correct and emotionally intelligent, resulting in deeper engagement and personalized learning experiences.

## 2. Related Works and Background

Virtual avatars can represent people online, enabling social interactions, collaborative learning, and secure communication. AI-powered educational avatars provide personalized training but confront issues such as disinformation and data privacy [4]. These avatars can be customized for specific material, languages, and teaching styles [5] and connected with user personalities for increased relevance [6]. Virtual tutors have been developed to improve cultural heritage education [7,8,9] and as helpful tools in case-based anatomy learning [10]. However, 19 limited studies have investigated AI avatars in immersive learning environments, exposing gaps in experience and research despite rising student interest in these approaches [11,12].

In healthcare, AI-driven avatars have demonstrated success in enhancing self-care knowledge [13], modeling clinical scenarios [14], supporting diabetic education via smartphones [15], and assisting Arab children with autistic spectrum disorder [16]. Trials are also being conducted to investigate their potential for acute coronary syndrome education [17] and reading skill enhancement in dyslexic youngsters [18]. Furthermore, virtual technologies assist impaired people in overcoming obstacles to online interaction [19].

A platform promotes collaborative learning and ethical decision-making in business education [20], whereas intelligent systems use AI and machine vision to enhance teaching efficacy [21]. Virtual humans enhance instructional environments with animations and decision-making capabilities [22], and research investigates the impact of avatar audio [23], interaction styles [24], and customization on user engagement and outcomes [25,26,27]. Furthermore, virtual simulations help with teacher preparation [28] and pre-service skill development [29], demonstrating the diverse potential of AI-driven avatars in education.

Recent studies have looked into many areas of avatar-based interaction and emotion recognition. Research [30] on 3D avatars demonstrates that integrating Blendshape adjustment, real-time facial capture, and text-driven lip synchronization can significantly improve emotion identification. Public attitudes toward virtual beings in China [31] focus on technology breakthroughs, virtual idols, and concerns about ethical difficulties and technological unemployment. Chatto, a conversational agent for elders [32], exhibits avatars’ potential for promoting social engagement and emotional well-being in ambient assisted living (AAL) environments. In immersive VR, avatars with pleasant facial expressions create trust, whereas negative ones reduce it [33]. Avatar personalization has also been proven to significantly impact emotional responses in VR, specifically happiness [34]. Furthermore, a study [35] on data-driven sign language interpreting avatars emphasizes its potential for increasing communication in the deaf community, giving significant insights into virtual signing systems. These findings highlight the relevance of avatar design, emotional expression, and personalization in improving user interaction across various apps.

Incorporating avatars capable of reading text with intonation and using text-to-speech (TTS) technology with expressive features dramatically improves the user experience by delivering a more natural and engaging audio interaction [36]. Notably, a study [37] has provided an intonation model adapted for the Kazakh language based on previous work in sentiment analysis [38,39,40,41,42,43,44] and the development of a morphological analyzer [45,46]. These fundamental efforts led to the development of this research, which intends to evaluate and enhance avatar-based learning systems by aligning linguistic structures with sentiment-driven expressions. Additionally, [47] determined the optimum technique for designing the interface of the Kazakh digital intelligent platform, which influenced the design and functionality of avatar-based systems. A complete semantic knowledge library of over 16,000 annotated words and phrases has been created [48], which includes a 5-point sentiment scale for nuanced sentiment identification. This hybrid model applies the semantic knowledge base to reliably measure sentiment orientation at the sentence and document levels, accounting for contextual variability, which is critical for practical natural language sentiment analysis. By classifying text into emotional categories—positive, neutral, and negative—the model enables avatars to demonstrate expressive behaviors, improving the learning experience in avatar-based systems.

## 3. Methods

**Figure 1:**
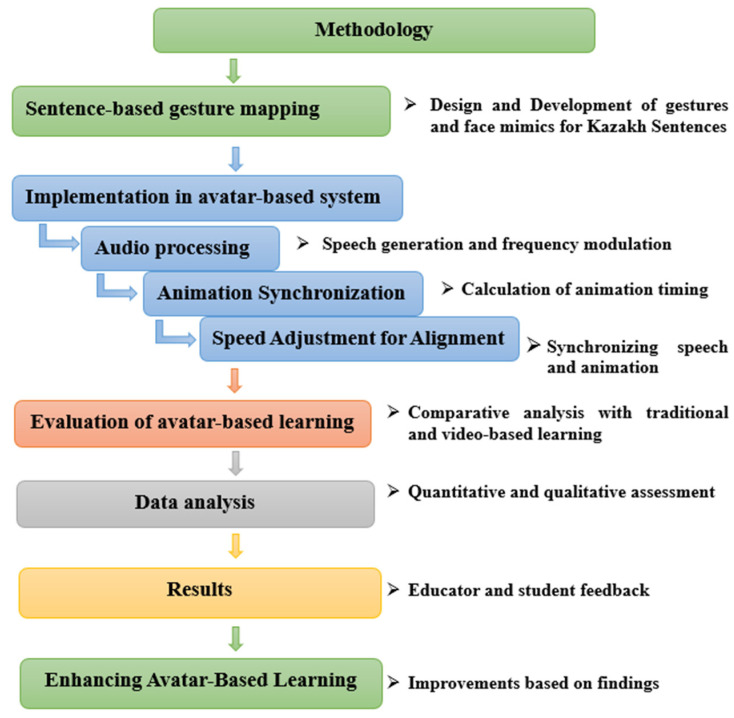
Research methodology.

The research methodology depicted in Figure 1 involves the creation and evaluation of an avatar-based learning system designed to align with Kazakh sentence structures. The process began with sentence-based gesture mapping, focusing on the design and development of gestures and facial mimics that correspond to Kazakh syntax, semantics, and cultural norms.

Following this, the gestures were implemented into the avatar-based system through the three key components of audio processing, which includes speech generation and frequency modulation for natural intonation; animation synchronization, which ensures the precise timing of animations to match speech patterns; and speed adjustment for alignment, which synchronizes speech and animation by adapting to varying speech tempos. The effectiveness of the system was evaluated through comparative analysis with traditional classroom learning and video-based lessons. This study measured knowledge gain, engagement, and perceived effectiveness across these learning modalities. A mixed-methods approach was applied for data analysis, incorporating quantitative assessments through pre- and post-test score comparisons using ANOVA alongside qualitative thematic analysis of participant responses. The results were shaped by educator and student feedback, identifying areas for refinement. Based on these insights, enhancements were made to the avatar-based system, improving gesture accuracy, synchronization, and usability to optimize its overall effectiveness. This structured methodology provides a strong foundation for developing and assessing innovative, technology-enhanced learning systems. This streamlined methodology offers a solid framework for creating and evaluating an innovative teaching system. 

### 3.1. Design of Sentence-Based Gesture Mapping for Kazakh Sentences

Sentence-based gesture mapping (SBGM) is recognized as a methodological framework particularly relevant in intelligent interactive systems, including avatar-based learning environments. This framework establishes associations between specific gestures and facial expressions and distinct sentence structures or categories, such as declarative, interrogative, and exclamatory sentences. The primary objective is to align non-verbal communication with the spoken content’s linguistic characteristics and emotional tone, thereby enhancing the naturalness and efficacy of interaction [49,50].

To ensure seamless interaction, gestures and facial expressions were meticulously synchronized with the avatar’s speech. This procedure required careful hand gesture and facial expression coordination with Kazakh phrases’ intonation and rhythmic patterns. Advanced animation software, such as Blender 3.6 (Amsterdam, Netherlands), created and optimized the synchronization between verbal and nonverbal elements.

Furthermore, a thorough investigation of Kazakh sentence structures was conducted to establish the best alignment of gestures and expressions with vocal communication. This analysis identified typical phrase structures and emotional expressions frequently seen in conversational Kazakh. A thorough study of Kazakh cultural norms and common emotional responses aided this mapping approach.

No existing solutions have been specifically developed for the Kazakh language that account for its syntactic structure when generating gesture animations. Most available virtual character gesture animation systems are designed for English or other widely spoken languages, where sentence structures differ significantly.

The proposed sentence-based gesture mapping model introduces an innovative approach by adapting gestures to the syntactic characteristics and word order of the Kazakh language. Unlike English, which follows a rigid subject-verb-object (SVO) structure, Kazakh exhibits greater flexibility, commonly employing subject-object-verb (SOV) and other structural variations. These syntactic differences influence intonation, pauses, and expressive elements in communication. By aligning gestures, facial expressions, and speech with the natural linguistic patterns of Kazakh, this model enhances the synchronization and realism of interactions in intelligent educational systems and avatar-based applications for Kazakh-speaking users. Table 1 depicts the gesture and facial expression models created for Kazakh sentence structures.

Previous research [37] presented a natural language processing framework and an intonational model for the Kazakh language, which served as the foundation for this investigation.

In the Kazakh language, the predicate is one of the key components of a sentence, and its form varies depending on the type of sentence. Interrogative sentences ask questions, and the predicate often involves a question particle or interrogative pronoun while exclamatory sentences express strong emotions like surprise, joy, or anger. The predicate is similar to that of declarative or interrogative sentences but is often accompanied by an exclamatory particle or intonation. Table 2 presents the combinations of gestures and facial expressions created for the predicate’s forms.

### 3.2. Implementation in Avatar Based System

**Figure 2:**
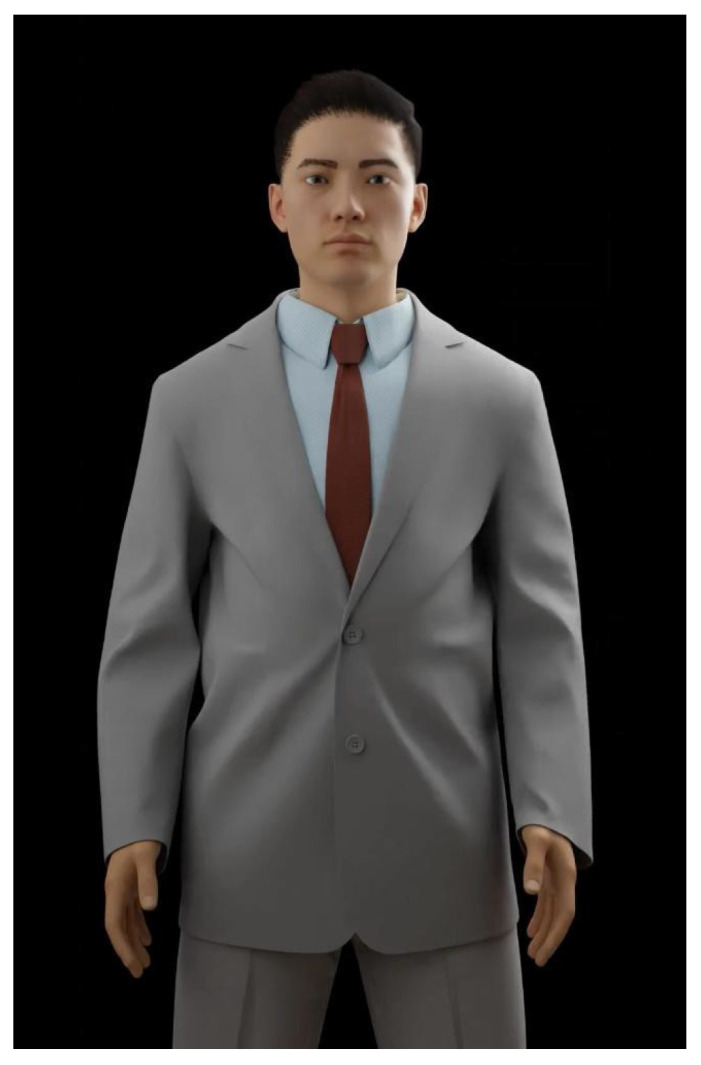
The 3D avatar which was created by Blender.

The designed motions and facial expressions were integrated into a 3D avatar, with movements and expressions modeled using Blender 3.6 to ensure fluidity and responsiveness. Blender is a sophisticated open-source 3D creation suite widely used across industries such as animation, modeling, simulation, and gaming. It provides comprehensive 3D content creation capabilities, including modeling, texturing, rigging, rendering, and compositing. Figure 2 illustrates a 3D avatar generated using Blender.

**Figure 3:**
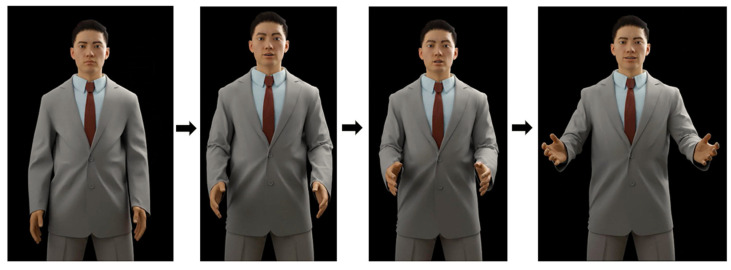
Selected frames illustrating the open-hand gesture in sentence structure 1.

For example, based on Sentence Structure 1, we will consider how the avatar performs gestures and facial expressions for the sentence. This sentence begins with the subject, and Figure 3 illustrates the corresponding use of gestures and facial expressions. According to Table 1, the avatar performs an open-hand gesture to draw attention to an object. Additionally, the avatar smiles to acknowledge the object positively and raises its eyebrows to express surprise or curiosity. Since it is not feasible to display the full animation sequence, Figure 1 presents key excerpts that illustrate the essential stages of executing the open-hand gesture. These selected frames effectively depict the transition and synchronization of facial expressions and gestures in alignment with the sentence structure.

The animations were saved in .mov format and dynamically assigned using Python 3.10 (Wilmington, DE, USA) scripts based on the input Kazakh sentence structure. This ensured that the avatar’s non-verbal communication was contextually relevant to the ongoing conversation.

This approach outlines how the gestures and facial expressions were designed and integrated, with a focus on aligning them with the cultural and linguistic characteristics of Kazakh sentences.

To integrate TTS output with avatar animations, ensuring synchronized audio-visual communication, the methodology involves the following three core components of audio generation and frequency modulation, calculation of animation timing, and speed adjustment for animation alignment.

#### 3.2.1. Audio Generation and Frequency Modulation

To generate speech, a local TTS engine (Microsoft SAPI 5.4, Redmond, WA, USA) with SSML was employed. SSML allowed control over the pitch, rate, and pauses, ensuring alignment with the syntactic structure of the sentence. The TTS system relied on the KazakhTTS2 dataset [51], which contains 271 h of speech from five speakers on a variety of topics. This corpus supports high-quality Kazakh TTS models, with evaluations averaging 3.6–4.2.

Each word in the input text is allocated a frequency range depending on its complexity score C, which is determined using the following formula [52,53,54,55]:

*C* = *a* · *S* + *b* · *L* + *c* · *W_POS_* (1)

where *a, b, c* are weighting factors assigned to each component; *S* is the number of syllables; *L*—number of characters; and *W_POS_*—weight to the type of word or parts of speech in a sentence.

The complexity score *C* is then transferred to a frequency range using the mapped frequency *F* formula based on these studies [56,57,58]:

(2) $$F\;=\;F_{\min}\;+\frac{\left(C-C_{s\;min}\right)}{C_{s\;max}-C_{s\;min}}\;F\cdot F_{\max}-F_{\min},$$

This formula ensures that words with lower complexity scores are mapped to the lower end of the frequency range, and words with higher complexity are transferred to the upper end.

The TTS output is supplemented with these frequency modulations via SSML, guaranteeing that the audio dynamically reflects word complexity.

#### 3.2.2. Calculation of Animation Timing

To synchronize the avatar’s motions with the TTS output, the system extracts the duration of each word from the generated audio. Word timings are used to elicit appropriate face emotions and gestures. Each animation corresponds to a certain word or phrase. TTS generates exact timing for each syllable, usually measured in milliseconds. Afterward, animations are designed to correspond with the calculated duration. For example, if a word has a duration of 500 ms, the corresponding animation is changed accordingly.

#### 3.2.3. Speed Adjustment for Animation Alignment

To synchronize avatar animations with TTS audio, a speed adjustment factor (SAF) is calculated to match the duration of animations to the spoken audio:

(3) $$\mathrm{SAF}=\frac{A_{ni}}{A_{ud}},$$

where $A_{ni}$ is the animation duration and $A_{ud}$ is the audio duration.

To synchronize avatar motions with TTS-generated speech, a speed adjustment factor (SAF) was developed and applied in Python. This approach included calculating the speed factor, resampling video frames, and altering playback speed to match the audio time. The MoviePy library was used to dynamically adjust the animation speed, resulting in precise alignment with the spoken content. Furthermore, Wav2Lip (Hyderabad, India) [59] was used to synchronize the avatar’s lip movements with the spoken text, which improved the realism of audiovisual communication. This approach ensures a smooth and synchronized audiovisual experience, improving the effectiveness of avatar-based learning.

This methodology ensures a fully synchronized audiovisual system in which avatar animations blend perfectly with TTS-generated sounds, resulting in a realistic and engaging user experience.

### 3.3. Evaluating Avatar-Based Learning Through Comparing with Other Methods

The primary goal of this research is to examine the effectiveness of three different teaching approaches, with a particular emphasis on evaluating the Avatar-based system (ABS) as a new approach to education. This system is a unique, interactive, AI-driven learning platform that simulates personalized education and engages students. This study compares it to traditional classroom learning (TCL) and video-recorded lessons (VRL) to identify its potential advantages and limitations in improving educational outcomes. This study evaluates the following hypotheses:

- The null hypothesis (H₀) states that there is no significant difference in learning outcomes between the three teaching methods.
- The alternative hypothesis (H₁) states that there are significant variations in learning outcomes.

To guarantee clarity and emphasis in this study, the variables have been carefully defined and classified. The independent variable is the teaching approach, which is classified into the three types of traditional classroom learning, video-recorded classes, and avatar-based systems. The dependent variable is learning effectiveness, which is assessed using post-test scores, retention rates, and the capacity to apply newly acquired abilities. To remove any confounding effects, several control factors were standardized, such as instructional content, session time, task difficulty, and participant demographics. These metrics verify that the observed disparities in outcomes are completely due to the educational techniques being evaluated.

A total of 90 participants were recruited through stratified sampling to assure demographic similarity. To ensure consistency, these individuals were divided into three groups of 30 students, each enrolled in a bachelor’s program at the same institution. All participants completed a standardized baseline test to assess their initial knowledge levels. This exam served as a reference point for monitoring subsequent learning gains.

A between-subjects design was used to compare the three instructional approaches. Group 1 participated in TCL through a live, face-to-face lecture. Group 2 learnt through VRL, which presented the same instructional information, whereas Group 3 used an ABS for interactive learning of the same topic. After the intervention, all participants were given a post-test that was identical to the pre-test. This test was used to determine knowledge gains and the efficacy of each teaching style. Furthermore, surveys were issued to solicit subjective input on participant engagement, contentment, and perceived learning efficacy.

This study gathered both quantitative and qualitative data to provide a comprehensive assessment of the teaching methods. The quantitative data include pre-test scores (Appendix A, Table A1) and post-test scores (Appendix B, Table A2), which were recorded for each participant in the three groups: TCL, VRL, and ABS. These tests measured students’ knowledge levels before and after the intervention. A statistical analysis was performed to evaluate learning improvements across the methods in the next section. Qualitative data, such as surveys, collected participant perceptions of satisfaction, engagement, and perceived learning efficacy. This systematic methodology ensures the reliability of the data and provides a comprehensive understanding of the efficiency of each teaching style.

An experiment with five educators was carried out to assess the effectiveness of the learning approaches. These instructors gave comments by comparing the three methods. To achieve a thorough assessment of the three learning modalities, a mixed-methods approach was used. This strategy merged quantitative data (ratings) with qualitative insights (open-ended survey questions). The combination of both methodologies provided for a more in-depth assessment of the findings, overcoming the limitations of a smaller sample size by collecting both numerical patterns and personal perspectives.

### 3.4. Data Analysis

Google Forms was used as the primary tool for developing and delivering surveys and tests to simplify the collection of feedback and test results. The platform’s user-friendly interface made it easy for participants to reply, and its connectivity with spreadsheets permitted seamless data export for additional analysis.

The data obtained from student assessments, questionnaires, and educator comments were analyzed using Python 3.10 and Microsoft Excel to ensure accuracy and complete insights. Python was used for the statistical analysis, which included calculating percentages, creating visualizations (such as bar charts and pie charts), and spotting trends in the data. Its powerful libraries, including Pandas 2.2.3 and Matplotlib 3.8.2, allowed for faster data processing and visualization. Excel was used to organize the raw data, complete preliminary analysis (e.g., averages, counts), and double-check the results for accuracy. Its user-friendly interface allowed for clear data representation and validation before moving on to deeper Python 3.10 analysis.

#### 3.4.1. Quantitative Data Analysis

The obtained data were subjected to rigorous statistical analysis [60] to establish the efficacy of each teaching method. To begin, the mean and standard deviation of each group’s pre- and post-test scores were computed to provide an overview of performance. Table 3 displays descriptive statistics for the quantitative analysis, which includes pre- and post-test scores.

These statistics illustrate the changes in test scores for each teaching method. They share information on the knowledge progress across groups by showing the variability (by standard deviations) and the magnitude of gains or losses in the learning effectiveness (via mean and range of gains).

The standard deviations for pre-test results were modest, indicating that baseline knowledge varied little within each group. The TCL group had a somewhat higher baseline than the other two. The mean post-test results rose for all groups. The TCL group showed the most progress, followed by the VRL group. The ABS showed minimal improvement. The range of gains shows that some individuals in the TCL approach improved significantly (up to +12), whilst the ABS made modest and consistent advances.

The TCL approach had the most variability in post-test scores and knowledge gains (*SD* = 3.87 and 5.35, respectively), indicating a wide range of outcomes for participants. Nonetheless, the ABS had the least variability (*SD* of gains = 1.46), indicating steady but modest performance.

Afterwards, the post-test results were used in a one-way analysis of variance (ANOVA) to see whether there are statistically significant variations in learning outcomes across the three teaching approaches. Table 4 summarizes the findings of the ANOVA analysis calculated using the [61] approach.

The F-statistics value is 4.6033. This number implies that the difference between group means is 4.603 times the variance within each group. The *p*-value of 0.0126 is smaller than the conventional significance level (e.g., 0.05), indicating a statistically significant difference in the means of the three groups. This shows that at least one of the learning strategies produces different results than the others.

However, the ANOVA test only tells us if there is a significant difference; it does not specify which groups are different. For that, a post-hoc test is required. ANOVA assumes that the data are approximately normally distributed, or the groups have equal variances (homogeneity of variances). To investigate the significant differences between the groups revealed in the ANOVA, we will perform a post-hoc test with Tukey’s honestly significant difference (*HSD*). It handles this by comparing all possible pairs of groups and determining where the disparities exist.

The Tukey *HSD* value is determined by the following formula [62]:

(4) $$\mathrm{HSD}\;=\;q\cdot \;\sqrt{\frac{MSW}{n}},$$

where *q* is the critical value from the studentized range distribution; *MSW* is the mean square within groups; and *n* is the number of observations in each group.

We need to look up the critical value (q) for α = 0.05, df_between = 2, and df_within = 87 in a Tukey’s *HSD* critical value table. *MSW* = 45.6445 from the ANOVA analysis table, *n* = 30. The result is *HSD* = 3.84 which means that any pairwise difference between the group means greater than 3.84 is considered statistically significant at the 0.05 level.

We now compare the differences between the group means to this *HSD* value to determine statistical significance if the TCL, VRL, and ABS groups have means of 4.2, 3, and 1.73, respectively.

Now, let us compute the pairwise differences.

1. TCL vs. VRL: ∣4.2 − 3∣ = 1.2 (not significant since 1.2 < 3.84);
2. TCL vs. ABS: ∣4.2 − 1.73∣ = 2.47 (not significant as 2.47 < 3.84);
3. VRL vs. ABS: ∣3 − 1.73∣ = 1.27 (not significant as 1.27 < 3.84).

Based on the HSD value of 3.84, none of the disparities between the group means exceeds this threshold, indicating that assuming the calculated means and values, there is no statistically significant difference between the groups in this investigation.

Next, metrics like Cohen’s *d* were used to estimate the practical significance of observed discrepancies. Cohen’s *d* calculates the effect size, or the amount of the difference between two groups. This adds to the statistical significance of the ANOVA test, which simply shows whether a difference exists, not how significant it is. The following formula determines Cohen’s *d* value [63]:

(5) $$d=\frac{{Mean}_{1}-{Mean}_{2}}{{SD}_{pooled}}$$

where ${SD}_{pooled}$ is the pooled standard deviation, computed as follows:

(6) $${SD}_{pooled}=\sqrt{\frac{\left(N_{1}-1\right)\cdot {SD}_{1}^{2}+(N_{2}-1)\cdot {SD}_{2}^{2}}{N_{1}+N_{2}-2}}$$

The results obtained from the formula indicate that the effect sizes differ between the learning strategies. Cohen’s *d* = 0.32 for TCL vs. VR suggests a moderate to medium effect size, implying tiny variations in learning outcomes that may have practical significance in some settings. In contrast, TCL vs. ABS gave Cohen’s *d* = 0.86, indicating a large effect size and emphasizing a significant practical advantage for the traditional classroom. Finally, the comparison between VRL and ABS resulted in Cohen’s *d* = 0.47, which is a medium effect size and indicates moderate differences, with video-recorded lectures showing some advantages over the avatar-based method.

#### 3.4.2. Qualitative Data Analysis

Following the intervention phase, participants (students) were surveyed to collect qualitative data. These surveys contained closed-ended Likert scale questions to capture broad trends and open-ended questions to elicit detailed participant input about their experiences with the assigned teaching method. The survey was designed to collect qualitative and quantitative feedback from participants on their experiences with the assigned learning technique. This input was designed to assess each method’s engagement, clarity, interactivity, and perceived efficacy and identify areas for improvement. The survey was administered online following the intervention phase. To promote honest feedback, participants completed the survey anonymously.

The teacher feedback survey sought professional perspectives from educators on the efficacy, engagement levels, and pedagogical worth of three learning approaches. These insights gave an experienced viewpoint to supplement the student-centered data.

## 4. Results

### 4.1. Student Feedback

The raw pre-test scores (Appendix A, Table A1) indicate that participants had varying levels of prior knowledge before the intervention. Some students, particularly in the VRL and ABS groups, had no initial understanding (score = 0), whereas the TCL group exhibited higher initial scores on average.

After the intervention, the post-test scores (Appendix B, Table A2) demonstrated improvements across all groups, with the highest individual gains observed in the VRL method. The ABS group also showed moderate improvements, while the TCL group exhibited mixed results.

The results of the analysis indicate some noteworthy facts about the efficacy of the three teaching methods. Pre-test results showed relatively minimal fluctuation among each group, indicating consistent baseline knowledge, albeit the TCL group had somewhat higher initial scores. Post-test scores increased across all groups, with the TCL group showing the most improvement, followed by the VRL and ABS groups. Notably, the TCL group exhibited significant individual knowledge gains, with some participants reaching +12, whereas the ABS group showed small but consistent gains. The TCL group had the most variability in outcomes (*SD* = 3.87 for post-test scores, *SD* = 5.35 for knowledge increases), indicating that this strategy elicited a wide range of participant reactions.

**Figure 4:**
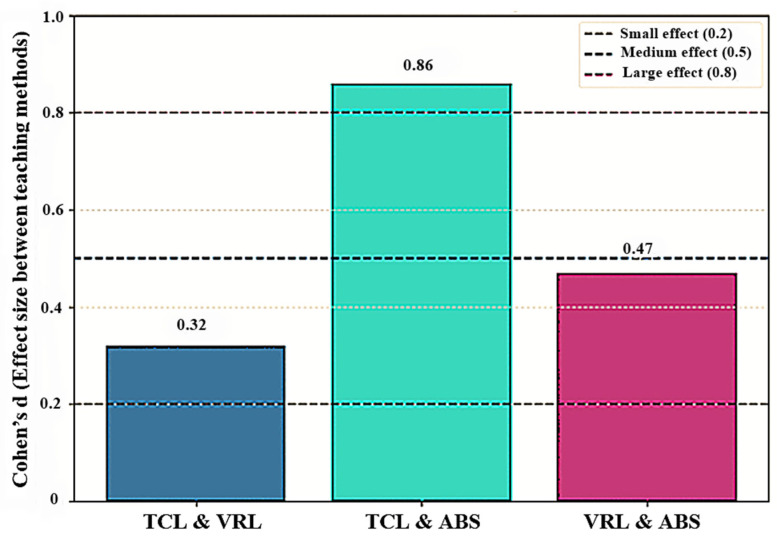
Effect sizes between teaching methods.

Statistical analysis using one-way ANOVA revealed a significant difference in post-test scores between groups (*F* = 4.6033, *p* = 0.0126). However, Tukey’s *HSD* post-hoc test revealed that none of the pairwise differences between group averages above the *HSD* threshold of 3.84, implying that no statistically significant differences existed between individual groups. The effect size analysis (Figure 4) demonstrates that while the ANOVA findings show statistically significant differences in learning outcomes between groups, the practical significance differs. The TCL approach has a significant practical advantage over the ABS, although the differences between VRL and the other two methods are less noticeable.

**Figure 5:**
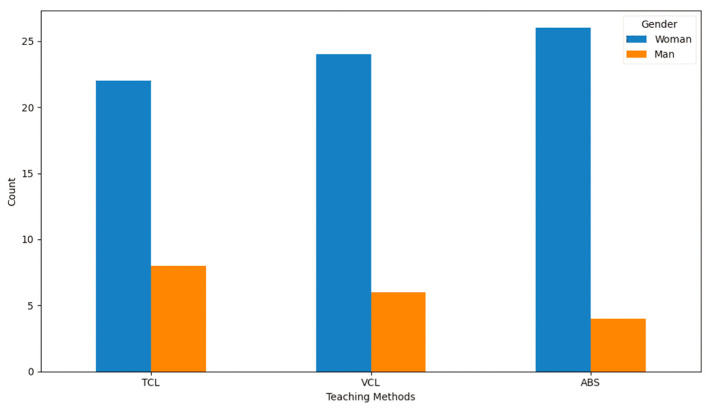
Gender distribution across teaching methods.

Participants in this study were chosen from the same age range and had the same educational level, resulting in a homogeneous sample. This uniformity removes external variables such as age or education, resulting in a consistent baseline for comparison. Although the participants are similar in age and educational background, the gender distribution among the three instructional modalities is shown in Figure 5.

The consistency of age and education assures that any reported differences in engagement, comfort, or perceived efficacy are more likely related to the teaching approach itself, rather than demographic variances.

While women consistently outnumber men across all approaches, the transfer from TCL to ABS shows a steady decline in male participants, indicating possible variations in comfort or preferences while switching to technology-driven learning environments.

**Figure 6:**
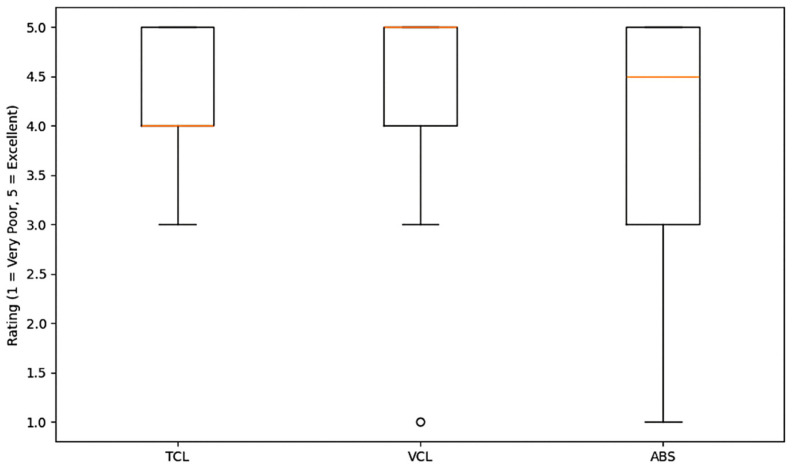
Overall learning experience ratings.

Moreover, survey analysis reveals that while moderate experience with online learning dominates across methods, ABS and VRL show higher variability in prior experience. Some students had extensive familiarity, while others reported minimal or no prior exposure. This gap in prior knowledge might impact comfort levels, engagement, or adaptability to new teaching methods. Overall, while students show some divergence in their ratings (Figure 6), ABS generally appears to be perceived more positively than TCL and VRL, with VRL having the widest range of feedback.

**Figure 7:**
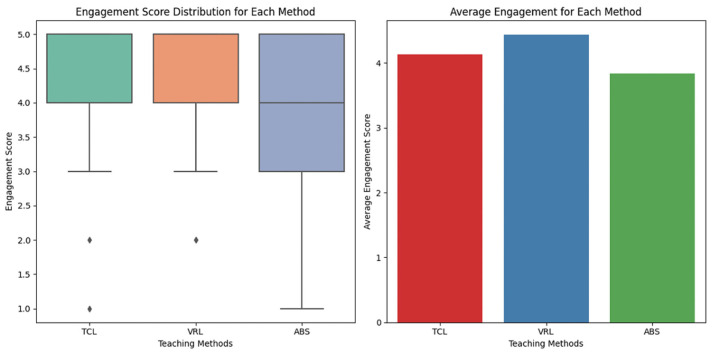
Engagement levels across teaching methods.

Figure 7 depicts a boxplot that visually compares the engagement scores for each teaching style, demonstrating the distribution and spread of ratings. Participants’ ratings ranged from one (poor engagement) to five (great engagement).

VRL was discovered to be the most engaging teaching style overall. TCL also performed well, albeit with little fluctuation. ABS elicited a wider range of responses, implying that it was engaging for some but not others.

**Figure 8:**
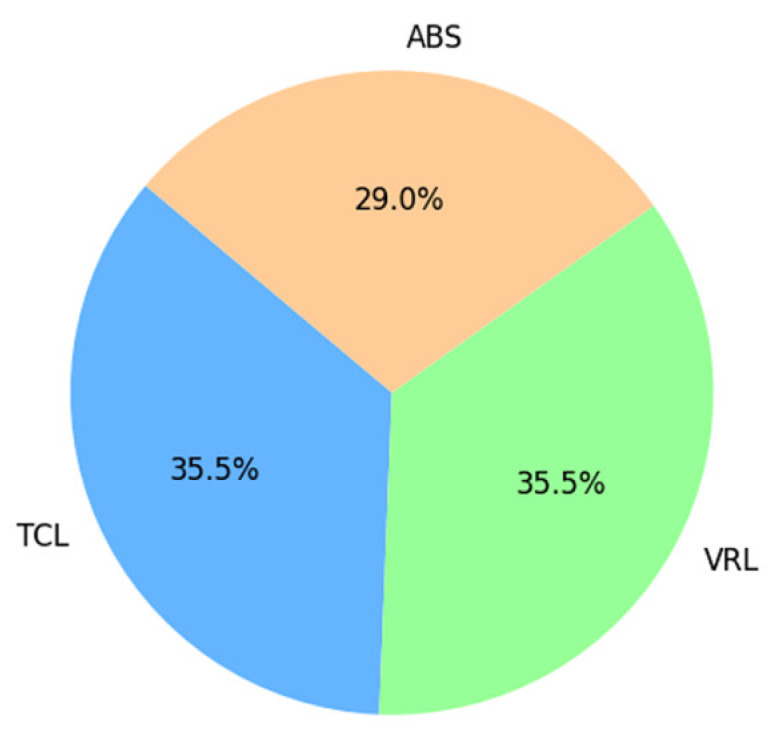
Average clarity and understandability across methods.

Figure 8 shows students’ ratings of how clear and understandable the topic was conveyed using the three different teaching approaches. The TCL and VRL techniques were equally effective in terms of clarity and understandability, accounting for 35.5% of the average distribution. The ABS technique obtained a lower average score, indicating that students perceived the knowledge conveyed through avatars slightly less clearly and intelligibly than the other ways.

**Figure 9:**
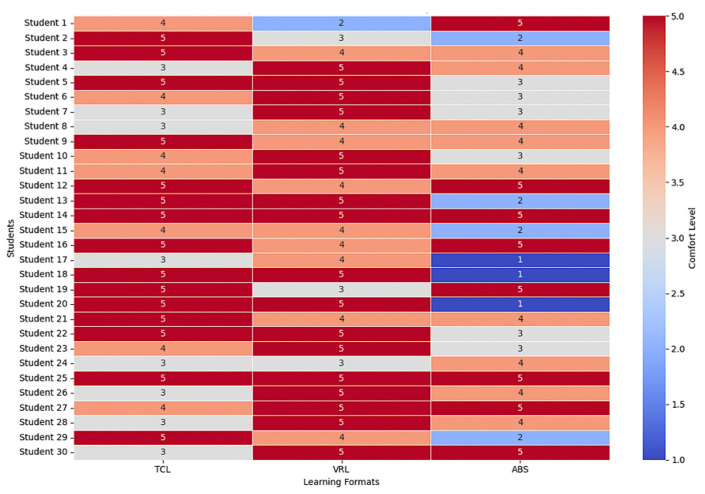
Heatmap of comfort level across learning formats.

A heatmap was used in Figure 9 to visually represent the student feedback on different teaching methods, making it easier to compare comfort across the methods. VRL tends to be the most generally comfortable strategy for students, followed by TCL, and ABS yields mixed results.

An examination of student responses to future learning approaches revealed unique trends. VRL was the most popular method, with the majority of students responding “Yes, definitely”. TCL also received a lot of good comments, with responses ranging from “Yes, definitely” to “Maybe”, showing that it has a lot of promise as a future learning approach. ABS, on the other hand, garnered mixed reviews. While a few students stated a strong preference (“Yes, definitely”), the majority skewed towards “Maybe”, with a significant percentage selecting “No, not at all”.

Table 5 is analyzed with percentages for each interactivity level across the three techniques. VRL has the highest number of highly interactive responses (46.67%), whereas ABS has the highest proportion of moderately interactive responses (56.67%). TCL has a very balanced distribution but includes a tiny fraction (6.67%) of not interactive replies, which are absent from VRL and ABS.

Students’ open-ended comments provided significant information on their preferred learning methods. Students valued the face-to-face interaction with teachers and peers, the organized atmosphere that encouraged focus and discipline, and the involvement in real-time conversations and collaborative activities. Regarding VRL, respondents emphasized the flexibility of accessing videos at their leisure, the option to pause, rewind, and repeat content for greater understanding, and the opportunity for autonomous study at their own speed. Additionally, students expressed that the ABS method was engaging, adaptable, and stimulating. It enabled them to learn at any time and in any location they preferred.

The survey’s open-ended question “What did you dislike or find challenging about this learning method?” yielded the following observations. Students complained that traditional schools’ set schedules and locations made it difficult for them to learn at their own pace or review information as needed. However, students noted that the VRL method lacked real-time connection with teachers and peers, making it impossible to ask questions or receive prompt feedback. Furthermore, several students found it difficult to stay engaged and motivated when watching videos, particularly for extended periods of time, which resulted in distractions or decreased concentration.

In addition, students provided suggestions for improving each learning method. For TCL, they recommended integrating multimedia and interactive digital tools to enhance engagement and cater to diverse learning styles. In VRL, students highlighted the lack of real-time interaction and suggested incorporating discussion forums, live Q&A sessions, and shorter, segmented videos to improve engagement. For ABS, they proposed refining speech expressiveness, adjusting pacing, and integrating a Kazakh accent to enhance cultural alignment and clarity. Additionally, many students emphasized the benefits of a hybrid learning model, combining structured classroom discussions with video-based and avatar-driven content to maximize flexibility and effectiveness. These insights offer valuable guidance for refining each approach and optimizing digital and traditional learning experiences.

### 4.2. Educator Feedback

**Figure 10:**
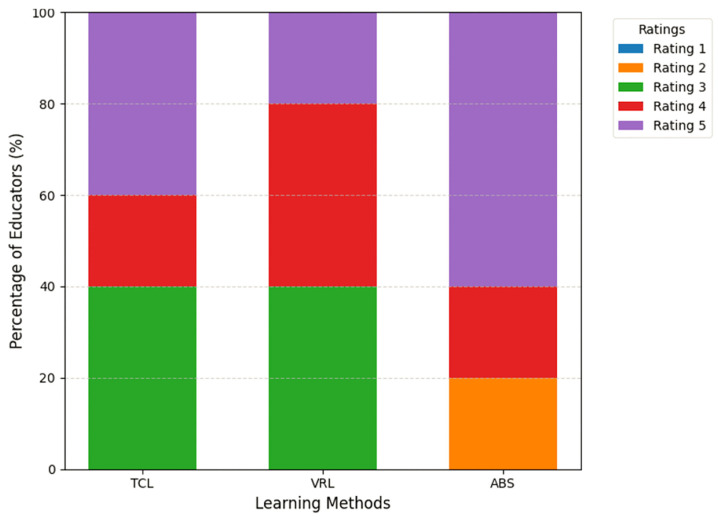
Educators’ ratings of learning methods’ effectiveness.

A total of 80% of educators reported 1–5 years of teaching experience, indicating that they are in their early professional stages and have new ideas on emerging teaching methods. The remaining 20% of the educators had 6–10 years of teaching experience, providing a more seasoned perspective when evaluating learning approaches. This distribution ensured a balanced mix of perspectives, drawing on insights from both the newer educators and those with more established teaching techniques. Figure 10 provides a stacked bar chart illustrating educators’ ratings of the three learning techniques’ effectiveness in improving student learning results. Each bar reflects the percentage of educators who provided a given rating (1–5) for each strategy.

ABS had the largest percentage of “highly effective” ratings, with 60% of educators choosing this choice. In comparison, only 40% and 20% of instructors regarded TCL and VRL as “highly effective”, respectively. However, ABS earned the highest percentage of minimal ratings, with 20% of educators rating it “slightly effective”. In contrast, neither TCL nor VRL earned any minimum scores (1 or 2), emphasizing their consistent perceived efficacy among instructors.

The average ratings for each approach, as determined by the Google Form analysis, are as follows: TCL—4.20, VRL—4.20, and ABS—4.40. These findings indicate that the ABS approach earned somewhat higher engagement ratings than TCL and VRL, which had equal average ratings. Participants were asked which technique they thought best supported differentiated education, which is described as responding to varied learning styles and capabilities. TCL has 60% interest out of 100%, demonstrating a significant belief in its capacity to enable individualized instruction. VRL has 40% interest out of 100%, indicating a lower preference than the other approaches. ABS likewise has 60% interest out of 100%, which matches TCL’s perceived efficacy in catering to different learning styles and abilities.

**Figure 11:**
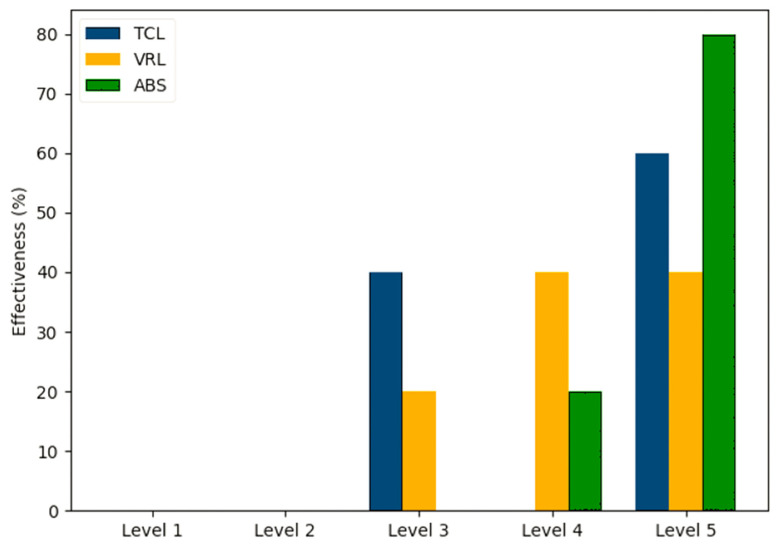
Effectiveness of methods in improving critical thinking and problem-solving skills.

Participants rated the effectiveness of three methods in improving students’ critical thinking and problem-solving skills on a scale from level 1 to level 5 in Figure 11. TCL’s effectiveness increases significantly between Levels 3 and 5, indicating a stronger reported influence at advanced levels. VRL has modest effectiveness, with consistent ratings at Levels 4 and 5. ABS is rated as the most successful method at Level 5, with significant improvement in ratings.

The following information was produced from an examination of responses obtained via a Google Form survey. Educators were asked to identify the strategy that they believe best promotes self-directed learning among pupils. The results, presented as percentages from 100%, are as follows: TCL—40%, VRL—60%, and ABS—60%. VRL and ABS appear to be similarly effective approaches for improving self-directed learning, with a stronger preference over traditional classroom learning. In addition, participants were asked how comfortable they were with incorporating technology (such as video classes or avatar systems) into their teaching. The findings, presented in percentages, show that rating 4 is 20%, while rating 5 is 80%.

The following observations are based on feedback from educators on the major strengths of each learning technique. Educators emphasized the need for face-to-face connection in cultivating instant feedback and a strong teacher–student relationship for TCL. They also commended VRL’s flexibility for both teachers and students, which allows lessons to be accessed at any time. They valued the ability to review content and the convenience of asynchronous learning. Educator feedback emphasized the ABS method’s engaging and interactive nature. They claimed that it boosts student motivation and enables for a more personal learning experience, especially for distant or self-paced students. Moreover, educators identified challenges related to limited flexibility in scheduling for TCL, as well as the potential difficulty in catering to the diverse learning needs of all students within a fixed timeframe. Some educators expressed concerns about the lack of real-time interaction for the VRL method, which can make it difficult to address student questions immediately. One of the challenges identified by educators regarding the ABS is the lack of a sense of an authentic teacher. While the system provides interactive and engaging content, some educators expressed concerns that the absence of a real human instructor may lead to a feeling of detachment or lack of personal connection.

Educators proposed numerous essential modifications, including expanding the authenticity of the avatar’s emotions, improving the emphasis in the pace of speech, and incorporating a Kazakh accent into the language to boost the ABS’s effectiveness. In response to the question about interest in using the ABS for their own teaching, 80% of educators indicated that they would be interested, while 20% expressed that they would not. This demonstrates a strong majority is open to integrating such technology into their teaching practices, though some reservations remain.

A comparative study was performed involving Syntheses [64,65], D-ID [66], and the suggested avatar-based intelligent interactive learning system to assess the functionalities of AI-enhanced avatar-based platforms in educational settings. The comparison emphasizes important aspects such as language support, customization of avatars, expressiveness of speech, interactivity, and deployment of the system. Table 6 provides a systematic summary of these platforms.

Existing platforms such as Synthesia and D-ID offer advanced AI-driven video generation and real-time interactions but are primarily designed for corporate training, marketing, and customer engagement. They lack deep integration with structured educational frameworks. Synthesia relies on pre-recorded video outputs, while D-ID supports real-time AI-driven interactions but lacks tailored educational adaptability.

A significant limitation of both is their insufficient support for the Kazakh language. While Synthesia supports over 140 languages, its Kazakh phonetic adaptation is underdeveloped. Similarly, D-ID’s Rosetta-1 model facilitates multilingual lip synchronization, but its accuracy for Kazakh phonemes remains unverified. Other related studies [4,5,6,7,8,9,10,11,12,13,14,15,16,17,18,19,20,21,22,23,24,25,26,27,28,29,30,31,32,33,34,35,36] also fail to address the linguistic and interactive needs of Kazakh-speaking learners fully.

In contrast, the intelligent ABS is specifically designed for Kazakh learners. It integrates custom sentence-based gestures and facial mimics aligned with Kazakh linguistic structure to ensure natural pronunciation, expressive speech delivery, and cultural relevance. Its real-time interactivity enables adaptive gestures, facial expressions, and speech intonation, enhancing engagement.

## 5. Discussion

The analysis of the teaching methods yielded several key insights into their effectiveness based on student performance and feedback. Pre-test scores showed minimal variability within each group, highlighting uniform baseline knowledge. However, the TCL group exhibited slightly higher initial scores. Post-test scores increased across all groups, with the TCL group showing the most significant improvements, followed by VRL and then ABS. The TCL group exhibited substantial knowledge gains but high variability in outcomes (SD = 5.35), while VRL showed moderate, balanced improvements. ABS demonstrated consistent but modest gains with the lowest variability (SD = 1.46), indicating its uniform yet limited effectiveness.

Statistical analysis using one-way ANOVA confirmed a significant difference in post-test scores among the groups (F = 4.6033, *p* = 0.0126). However, Tukey’s HSD post-hoc test indicated that pairwise differences between group means did not exceed the threshold of 3.84, suggesting no statistically significant differences between specific groups. While the ANOVA results indicate a significant overall difference among the teaching methods, the lack of statistically significant pairwise differences identified by Tukey’s HSD suggests that the variability within groups may have diluted the observed effects. This limitation, combined with the relatively small sample sizes, may have impacted on the statistical power of the analysis. While stratified sampling was used to ensure group representativeness, the sample size of 90 individuals (30 each group) remained a restriction. A greater sample size might improve the statistical results’ reliability and generalizability, potentially lowering the impact of individual differences on the conclusions. Future research should include increasing the sample size to improve the validity of the findings and investigating how different learner demographics influence the effectiveness of ABS, TCL, and VRL techniques.

Nevertheless, effect size analysis suggests that TCL offers a meaningful practical advantage over the ABS. This finding implies that students in TCL may have benefited from direct instructor interaction, a structured learning environment, or other pedagogical factors that contributed to improved learning outcomes. The relatively small effect size differences between VRL and the other methods indicate that VRL may serve as a viable alternative, particularly in scenarios where traditional classroom instruction is not feasible.

The lower performance of ABS may be attributed to its novelty, suggesting that students may require additional time to adapt to this learning approach. However, the findings also highlight the need for further refinement of ABS, including enhancements in interactivity, the integration of adaptive feedback mechanisms, and the incorporation of sentiment-driven gestures and facial expressions. These improvements could potentially bridge the gap between avatar-based learning and traditional instructional methods. Further research should examine the influence of learner engagement, cognitive load, and instructional design on the practical effectiveness of various teaching methodologies to optimize the implementation of intelligent interactive learning systems. While this study focusses on short-term evaluations of ABS using immediate post-test findings, knowing its long-term influence is an important area for future research. Assessing long-term knowledge retention, learner engagement, and flexibility would reveal more about the system’s effectiveness.

The deployment of ABS in educational settings does not necessitate extensive hardware. The animations and interactions can be run on standard computers or web-based platforms. While tools like Blender and Wav2Lip are freely available, their effective use—particularly for rendering and real-time interaction—may require computational resources such as mid-to-high-end GPUs. However, once animations are pre-rendered, the system operates efficiently on standard hardware, ensuring accessibility for various educational institutions without significant resource constraints.

The data presented in this study reveal valuable insights into educators’ perceptions of three distinct learning methods. The majority of educators (80%) are relatively inexperienced in their teaching careers, offering fresh perspectives on emerging methods, while 20% have more experience, contributing a balanced viewpoint.

When it comes to differentiated instruction, both ABS and TCL were equally favored (60%), indicating their perceived effectiveness in addressing diverse learning styles. However, for critical thinking and problem-solving, ABS emerged as the most effective method at the advanced level, with TCL showing a more significant impact at higher levels.

The analysis of self-directed learning revealed a strong preference for VRL and ABS, with both methods seen as more conducive to student autonomy compared to traditional classroom learning. Furthermore, educators expressed a high level of comfort with integrating technology, especially video lessons and avatar systems, into their teaching.

In terms of strengths, TCL was praised for its face-to-face interaction, VRL for its flexibility, and ABS for its engaging and interactive nature. However, educators highlighted challenges related to the lack of real-time interaction in VRL and the absence of a “real” teacher in ABS. To improve ABS, educators recommended enhancing the avatar’s emotional authenticity and incorporating a Kazakh accent to strengthen the learning experience.

Finally, the survey indicates a good response to incorporating ABS into teaching techniques, with 80% of educators showing interest in applying it, while certain worries remain. The findings show the need for additional ABS improvement to meet educator concerns and maximize its usefulness in modern classroom environments.

Based on participant feedback and perceived constraints, we chose to improve the ABS by incorporating sentiment-driven gestures and facial mimics. This attempts to create a more empathic and engaging learning environment, potentially boosting its effectiveness. This finding is consistent with the accumulating evidence that emotionally intelligent systems generate improved engagement and learning results.

## 6. Sentiment-Driven Development

The semantic knowledge base from the research [48] incorporates a 5-point sentiment scale, enabling the detection of nuanced sentiment. The semantic base also considers contextual variability, which is essential for natural language sentiment analysis. Building on this, the sentiment analysis model identifies emotions in input text. By categorizing the text into these emotional groups, the model supports the generation of expressive behaviors in avatars. To enhance the responsiveness and engagement of the avatar in the learning system, facial expressions and gestures are dynamically adapted based on the sentiment analysis results. The system utilizes a 5-point sentiment scale (−2 to +2) to categorize emotions into the five levels of very negative, negative, neutral, positive, and very positive. Each sentiment category is associated with a specific set of facial expressions and gestures in Table 7, designed to naturally reflect the emotional tone of the analyzed text.

By combining linguistic structure alignment with sentiment analysis, the avatar generates context-aware responses that are both grammatically accurate and emotionally resonant. For example, while linguistic structure guides the avatar to emphasize key sentence elements, sentiment analysis enriches this response with appropriate emotional expressions and gestures. This dual-layered approach ensures that the avatar’s reactions consider both the meaning and emotional tone of the input text. For instance, if a learner inputs a positive question, the avatar smiles, raises its eyebrows (to indicate attentiveness), and uses open gestures while providing an encouraging answer. For a negative statement, the avatar furrows its brows, tilts its head empathetically, and adopts a slower, softer tone while delivering feedback.

The avatar’s behavior adapts in real-time, ensuring responses that feel natural and contextually appropriate. The integration of linguistic structure ensures the avatar’s gestures and expressions emphasize key grammatical and semantic elements, while sentiment analysis provides the emotional depth needed for a truly engaging interaction. This approach results in an emotionally intelligent avatar capable of fostering deeper engagement and support within the learning environment. It not only enhances learner motivation but also builds a more empathetic and personalized educational experience.

A lexicon-based sentiment analysis approach will be applied to match the avatar’s motions and facial expressions to the emotional tone of the spoken information. To assess a sentence’s overall sentiment, the analysis uses tokenization, lemmatization, lexicon matching, and contextual aggregation. The avatar selects appropriate actions and facial expressions based on the derived sentiment score. To ensure temporal synchronization, animations are assigned in accordance with sentence structure and speech duration, using a SAF in MoviePy to adjust animation speed, Wav2Lip for precise lip synchronization, and blended animation sequences to smooth transitions between sentiment shifts. This strategy improves the expressiveness of the avatar, increasing engagement and realism in avatar-based learning.

## 7. Conclusions

This study underscores the transformative potential of the ABS method in modern education by comparing it to TCL and VRL. While TCL demonstrated the highest knowledge gains and VRL provided flexibility, ABS emerged as a consistent and promising tool for fostering critical thinking, self-directed learning, and personalized educational experiences.

However, this study also revealed key limitations in ABS, particularly the lack of emotional alignment and cultural authenticity. These challenges emphasize the need for enhancements to make ABS more engaging and relatable for learners. To address these gaps, we propose integrating sentiment-driven development into ABS. By leveraging a semantic knowledge base and hybrid sentiment analysis model for Kazakh texts, future systems can dynamically adjust gestures, facial mimics, and expressions to align with linguistic structure and emotional tone.

This sentiment-driven method attempts to elevate ABS beyond basic accuracy to emotional intelligence, resulting in deeper learner engagement and better outcomes. Sentiment-driven animations will be refined by increasing transition handling between emotional states, resulting in smoother, more natural avatar movements. Furthermore, including culturally relevant characteristics such as a Kazakh accent and adaptive learning methods would improve the system’s responsiveness and relatability.

As educators increasingly adopt technology-driven methods, these advances position ABS as a pivotal tool in modern teaching, capable of addressing diverse learning needs. Future work will focus on evaluating the impact of these enhancements on learner engagement and performance, as well as conducting long-term studies to assess the system’s effectiveness over extended periods. In the long term, understanding how ABS influences knowledge retention, sustained motivation, and learning adaptability will provide deeper insights into its role in modern education. These findings will lay the groundwork for adopting emotionally intelligent avatar-based systems in education.

## Figures and Tables

**Table 1:** Models of gestures and face mimics for Kazakh sentence structures.

Word Class of the Sentence Structure	Gesture	Face Mimics
*Sentence structure 1:*		
Subject	open hand gesture to draw attention to an object.	a smile to positively acknowledge an object; raise eyebrows to express surprise or curiosity about an object.
⋯	⋯	⋯
*Sentence structure 20:*		
*Attributive*	to show both palms with fingers naturally spread around the abdomen and concentrate on explaining the object.	raise eyebrows and slight smile as characteristic signs of attributive gestures.
*Object*	to bring the fingertips of both hands together towards the abdomen, denoting the object.	slight smile, tilt the head slightly to the right which typically conveys a sense of gentle curiosity, mild amusement, or a relaxed, approachable demeanor.
*Subject*	to emphasize subject priority, align the fingers of both hands in a straight line at the abdomen.	slight smile and slightly raised eyebrows which convey a blend of calmness, mild curiosity, or gentle amusement. This combination suggests a relaxed but attentive or interesting expression.
*Adverbial modifier*	the position of the subject point is defined in action methods, and the gesture is retained in place.	to imagine himself in motion with a mixed gaze to convey the image of the action
Predicate	to indicate the end of an action, return the hands to their neutral initial posture.	smile, nod slightly in agreement or gratitude, and then slowly turn avatar’s gaze to the left, indicating that the conversation has come to an end

**Table 2:** Models of gesture and face mimics for interrogative and exclamatory forms of sentence.

Predicate	Gesture	Face Mimics
Interrogative form	raising one eyebrow to show doubt;	to gaze with head tilted to the side to show curiosity or doubt;
Exclamatory form	to create a visual picture of the exclamatory sentence’s dynamic structure using sweeping hand movements (left).	using a slight smile to indicate the relationship between the subject and the predicate.

**Table 3:** Descriptive statistics.

Group	Pre-Test Mean ± *SD*	Post-Test Mean ± *SD*	Knowledge Gain Mean ± *SD*
TCL	2.73 ± 2.32	4.20 ± 3.87	1.47 ± 5.35
VRL	1.93 ± 1.34	3.00 ± 3.67	1.07 ± 3.78
ABS	1.47 ± 1.17	1.73 ± 1.14	0.27 ± 1.46

**Table 4:** ANOVA summary.

Source	Degrees of Freedom (*DF*)	Sum of Squares	Mean Square (*MS*)	*F-stat*	*p*-Value
Between groups	2	91.2889	45.6445	4.6033	0.0126
Within groups	87	862.6667	9.9157		
Total	89	953.9556			

**Table 5:** Breakdown of interactivity levels as percentages for TCL, VRL, and ABS.

Interactivity	TCL (%)	VRL (%)	ABS (%)
Highly Interactive	33.33%	46.67%	26.67%
Moderately Interactive	53.33%	46.67%	56.67%
Slightly Interactive	6.67%	6.67%	16.67%
Not Interactive	6.67%	0.00%	0.00%

**Table 6:** Comparison of AI-driven avatar-based platforms.

Feature	Synthesia	D-ID	Avatar-Based System
Platform overview	A video generation platform powered by AI features over 230 avatars, enabling users to produce videos in more than 140 languages. It is ideal for corporate training, marketing, and creating explainer videos.	AI-driven video and audio manipulation platform enabling dynamic avatar-based interaction. It is primarily used for customer support, real-time digital human applications, and content automation.	Web-based intelligent interactive learning system designed specifically for the Kazakh language, integrating speech synthesis, avatar animation, and sentiment-driven interactions to improve engagement.
Language support	Over 140 languages are supported, but the availability of support for the Kazakh language is limited or lacks native phonetic adjustments.	Offers support for various languages; however, Kazakh is not specifically mentioned, and the precision of phonetic representation is unclear.	Created uniquely for the Kazakh language, guaranteeing precise phonetic articulation, authentic intonation, and cultural relevance for those learning.
Avatars and customization	Offers a range of pre-designed avatars, with limited ability to modify facial expressions and gestures dynamically.	Provides customizable avatars, allowing users to manipulate visual appearance and emotional expressions to some extent.	Custom-designed avatar, incorporating sentence-based gestures and facial mimics optimized for Kazakh linguistic structure.
Speech expressiveness	AI-generated speech with preset intonation. Limited Customization over voice modulation.	AI-driven voice synchronization lacks real-time intonation control tailored for specific linguistic needs.	Applies sentence-based intonation mapping for Kazakh, ensuring natural sounding speech and expressive vocal delivery for better comprehension.
Interactivity	Mainly consists of pre-recorded videos, which do not offer real-time engagement or user interaction.	It focuses on real-time AI-driven conversations but lacks deep integration with structured educational content.	An engaging learning environment in which the avatar adjusts its gestures, facial expressions, and vocal intonation in real-time according to the material and user engagement.
Facial and gesture animation	GAN- and CNN-based facial animation; natural expressions but limited emotional variation. Gesture animation with minimal head movement and no hand gestures.	AI-generated facial motion with subtle expressions. Gesture animation with limited head movement, no hand gestures.	Custom sentence-based expressions using Blender 3.6. Full-body gestures dynamically linked to speech.
Lip synchronization	Utilizes a proprietary AI system that leverages machine learning and deep learning techniques to achieve photorealistic facial synthesis. However, that is not optimized for Kazakh speech articulation.	It employs a proprietary model known as Rosetta-1, which integrates AI capabilities for video translation, voice cloning, and lip-syncing. This model enables the synchronization of lip movements with translated audio while preserving the original speaker’s voice characteristics. But that is not explicitly trained for Kazakh phonemes.	Integrated with Wav2Lip for lip synchronization, ensuring accurate lip movements corresponding to Kazakh text-to-speech (TTS) output.
Security and compliance	SOC 2 and GDPR compliant, ensuring data privacy.	Employs SSL encryption (TLS 1.3) along with the automatic removal of temporary data to ensure security.	Custom data privacy controls aligned with organizational security policies.
System deployment and accessibility	Web-based, accessible via browser on Windows, macOS, Linux, requiring an internet connection.	Web-based, accessible on various platforms but dependent on cloud-based AI processing.	Web-based and deployable on institutional servers, supporting both online and offline modes for improved accessibility in educational environments.

**Table 7:** Mapping sentiment scores to expressions and gestures.

Sentiment Score	Description	Facial Expressions	Gestures
−2 (Very negative)	At this extreme, the avatar conveys strong dissatisfaction or frustration through:	Deeply furrowed brows, tightly pressed lips, and narrowed eyes	A slow shake of the head, crossed arms, and a slight backward lean to indicate withdrawal.
−1 (Negative)	The avatar displays subtle concern or disappointment through:	A slight frown, raised inner eyebrows, and slightly drooping eyes.	Slow, minimal hand movements, a slight downward head tilt, and simulated sigh gestures.
0 (Neutral)	The avatar maintains a composed and attentive demeanor through:	Relaxed features, natural resting eyebrows, and minimal eye movement.	Subdued hand movements, a small nod for attentiveness, and an open hand position to convey neutrality.
+1 (Positive)	Positive emotions are represented with a friendly and encouraging presence through:	A soft smile slightly raised outer eyebrows, and bright eyes.	Open hand gestures, a slight forward lean, and subtle applause.
+2 (Very Positive)	The avatar displays animated joy and enthusiasm through:	A broad smile showing teeth, lifted cheeks, and fully raised eyebrows.	A clear thumbs-up, animated waving, and open arms to symbolize warmth and happiness.

## Data Availability

Data are contained within the article.

## Appendix A

**Table A1:** The pre-test scores for each participant in the groups.

Participants	Traditional Classroom	Video-Recorded	Avatar-Based
Student 1	2	0	2
Student 2	2	2	2
Student 3	2	0	2
Student 4	2	2	0
Student 5	2	4	2
Student 6	2	4	2
Student 7	2	2	2
Student 8	2	2	2
Student 9	0	2	4
Student 10	2	4	2
Student 11	2	2	4
Student 12	2	2	2
Student 13	0	2	2
Student 14	2	0	0
Student 15	2	4	2
Student 16	2	2	2
Student 17	0	2	2
Student 18	0	0	0
Student 19	2	0	0
Student 20	2	0	0
Student 21	4	0	2
Student 22	8	4	0
Student 23	8	2	0
Student 24	8	2	2
Student 25	4	2	0
Student 26	2	4	2
Student 27	8	2	0
Student 28	2	2	2
Student 29	4	2	2
Student 30	2	2	0

## Appendix B

**Table A2:** The post-test scores for each participant in the groups.

Participants	Traditional Classroom	Video-Recorded	Avatar-Based
Student 1	4	2	2
Student 2	8	2	2
Student 3	2	16	4
Student 4	2	0	2
Student 5	6	2	2
Student 6	6	4	2
Student 7	12	2	2
Student 8	0	2	2
Student 9	2	0	2
Student 10	6	0	0
Student 11	2	2	2
Student 12	8	2	0
Student 13	12	2	0
Student 14	10	2	2
Student 15	2	6	2
Student 16	0	2	2
Student 17	10	2	2
Student 18	12	0	2
Student 19	0	0	0
Student 20	2	4	0
Student 21	4	4	2
Student 22	0	14	0
Student 23	0	0	2
Student 24	2	2	2
Student 25	2	4	2
Student 26	2	6	2
Student 27	2	2	4
Student 28	2	2	4
Student 29	4	0	2
Student 30	2	4	0

## References

[1] Machneva M., Evans A.M., Stavrova O. (2022). Consensus and (lack of) accuracy in perceptions of avatar trustworthiness. Comput. Human Behav..

[2] Hara C.Y.N., Goes F.d.S.N., Camargo R.A.A., Fonseca L.M.M., Aredes N.D.A. (2021). Design and evaluation of a 3D serious game for communication learning in nursing education. Nurse Educ. Today.

[3] Ji H., Han I., Ko Y. (2023). A systematic review of conversational AI in language education: Focusing on the collaboration with human teachers. J. Res. Technol. Educ..

[4] Fink M.C., Robinson S.A., Ertl B. (2024). AI-based avatars are changing the way we learn and teach: Benefits and challenges. Front. Educ..

[5] Mageira K., Pittou D., Papasalouros A., Kotis K., Zangogianni P., Daradoumis A. (2022). Educational AI Chatbots for Content and Language Integrated Learning. Appl. Sci..

[6] Shumanov M., Johnson L. (2021). Making conversations with chatbots more personalized. Comput. Human Behav..

[7] Campitiello L., Beatini V., Di Tore S. Non-player Character Smart in Virtual Learning Environment: Empowering Education Through Artificial Intelligence. Proceedings of the WAILS 2024.

[8] Gao K., Lee D.Y. (2024). Exploring the Effect of Youth Cultural Heritage Education Using the Metaverse Platform: A Case Study of “Pingyao Ancient City”. IEEE Access.

[9] Xie Y., Su M., Nie X., Li X. Digital Storytelling of Intangible Cultural Heritage: A Multimodal Interactive Serious Game for Teaching Folk Dance. Proceedings of the 12th International Conference, C&C 2024, Held as Part of the 26th HCI International Conference, HCII 2024.

[10] Chheang V., Sharmin S., Márquez-Hernández R., Patel M., Rajasekaran D., Caulfield G., Kiafar B., Li J., Kullu P., Barmaki R.L. (2024). Towards Anatomy Education with Generative AI-based Virtual Assistants in Immersive Virtual Reality Environments. Proceedings of the 2024 IEEE International Conference on Artificial Intelligence and eXtended and Virtual Reality (AIxVR).

[11] Dai C.P., Ke F., Pan Y., Moon J., Liu Z. (2024). Effects of Artificial Intelligence-Powered Virtual Agents on Learning Outcomes in Computer-Based Simulations: A Meta-Analysis. Educ. Psychol. Rev..

[12] Nagadeepa C., Pushpa A., Mukthar K.P.J., El Khoury R., Alareeni B. (2023). Are You Ready to Take Avatar in Virtual Classroom—Metaverse in Education from Student’s Perspective. How the Metaverse Will Reshape Business and Sustainability.

[13] Du H., Wonggom P., Burdeniuk C., Wight J., Nolan P., Barry T., Nesbitt K., Clark R.A. (2020). Development and feasibility testing of an interactive avatar education application for education of patients with heart failure. Br. J. Card. Nurs..

[14] Thompson J., White S., Chapman S. (2020). Interactive Clinical Avatar Use in Pharmacist Preregistration Training: Design and Review. J. Med. Internet Res..

[15] Bishop E., Allington D., Ringrose T., Martin C., Aldea A., García-Jaramillo M., León-Vargas F., Leal Y., Henao D., Gómez A.M. (2023). Design and Usability of an Avatar-Based Learning Program to Support Diabetes Education: Quality Improvement Study in Colombia. J. Diabetes Sci. Technol..

[16] Sweidan S.Z., Almawajdeh S.K., Khawaldeh A.M., Darabkh K.A. (2024). MOLHEM: An innovative android application with an interactive avatar-based chatbot for Arab children with ASD. Educ. Inf. Technol..

[17] Ellis T., Cheng S., Zecchin R., Zwack C., Hyun K., Zhang L., Gallagher R., Clark R., Redfern J. (2023). Effect of an avatar-based discharge education application on knowledge and behaviour in people after acute coronary syndrome: Protocol for a pragmatic prospective randomised controlled trial. BMJ Open.

[18] Amiri O., Shahab M., Mohebati M.M., Miryazdi S.A., Amiri H., Meghdari A., Alemi M., Pouretemad H.R., Taheri A. Virtual Reality Serious Game with the TABAN Robot Avatar for Educational Rehabilitation of Dyslexic Children. Proceedings of the 15th International Conference, ICSR 2023.

[19] Guo Z., Wang Z., Jin X. (2021). “Avatar to Person” (ATP) Virtual Human Social Ability Enhanced System for Disabled People. Wirel. Commun. Mob. Comput..

[20] Hu Y.H., Yu H.Y., Tzeng J.W., Zhong K.C. (2023). Using an avatar-based digital collaboration platform to foster ethical education for university students. Comput. Educ..

[21] Hu Y., Li Q., Hsu S. (2022). Interactive visual computer vision analysis based on artificial intelligence technology in intelligent education. Neural Comput Appl..

[22] Laureano-Cruces A.L., Sánchez-Guerrero L., Ramírez-Rodríguez J., Ramírez-Laureano E. (2022). Intelligent Interfaces: Pedagogical Agents and Virtual Humans. Int. J. Intell. Sci..

[23] Kao D., Ratan R., Mousas C., Magana A.J. (2021). The Effects of a Self-Similar Avatar Voice in Educational Games. Proc. ACM Hum. Comput. Interact..

[24] Yuan Q., Gao Q. (2024). Being There, and Being Together: Avatar Appearance and Peer Interaction in VR Classrooms for Video-Based Learning. Int. J. Hum. Comput. Interact..

[25] Pang H., Tang S., Han J.Y., Fung F.M. (2023). Exploring the Use of an Avatar-Based Online Platform to Facilitate Social Interaction in Laboratory Sessions. J. Chem. Educ..

[26] Segaran K., Mohamad Ali A.Z., Hoe T.W. (2021). Does avatar design in educational games promote a positive emotional experience among learners?. E-Learn. Digit. Media.

[27] Lim C., Ratan R., Foxman M., Meshi D., Liu H., Hales G.E., Lei Y.S. (2024). An Avatar’s worth in the metaverse workplace: Assessing predictors of avatar customization valuation. Comput. Hum. Behav..

[28] Berg C., Dieker L., Scolavino R. (2023). Using a Virtual Avatar Teaching Simulation and an Evidence-Based Teacher Observation Tool: A Synergistic Combination for Teacher Preparation. Educ. Sci..

[29] Lindberg S., Jönsson A. (2023). Preservice Teachers Training with Avatars: A Systematic Literature Review of “Human-in-the-Loop” Simulations in Teacher Education and Special Education. Educ. Sci..

[30] Mukashev D., Kairgaliyev M., Alibekov U., Oralbayeva N., Sandygulova A. (2021). Facial expression generation of 3D avatar based on semantic analysis. Proceedings of the 2021 30th IEEE International Conference on Robot & Human Interactive Communication (RO-MAN).

[31] Li S., Chen J. (2024). Virtual human on social media: Text mining and sentiment analysis. Technol. Soc..

[32] Mendes C., Pereira R., Ribeiro J., Rodrigues N., Pereira A. Chatto: An emotionally intelligent avatar for elderly care in ambient assisted living. Proceedings of the International Symposium on Ambient Intelligence.

[33] Luo L., Weng D., Ding N., Hao J., Tu Z. (2023). The effect of avatar facial expressions on trust building in social virtual reality. Vis. Comput..

[34] Radiah R., Roth D., Alt F., Abdelrahman Y. (2023). The influence of avatar personalization on emotions in vr. Multimodal Technol. Interact..

[35] Imashev A., Oralbayeva N., Kimmelman V., Sandygulova A. A user-centered evaluation of the data-driven sign language avatar system: A pilot study. Proceedings of the 10th International Conference on Human-Agent Interaction.

[36] Ukenova A., Bekmanova G. (2023). A review of intelligent interactive learning methods. Front. Comput. Sci..

[37] Amangeldy N., Ukenova A., Bekmanova G., Razakhova B., Milosz M., Kudubayeva S. (2023). Continuous Sign Language Recognition and Its Translation into Intonation-Colored Speech. Sensors.

[38] Yergesh B., Bekmanova G., Sharipbay A. (2019). Sentiment analysis of Kazakh text and their polarity. Web Intell..

[39] Bekmanova G., Yergesh B., Sharipbay A. (2021). Sentiment analysis model based on the word structural representation. Proceedings of the Brain Informatics: 14th International Conference, BI 2021.

[40] Bekmanova G., Yergesh B., Sharipbay A., Mukanova A. (2022). Emotional speech recognition method based on word transcription. Sensors.

[41] Yergesh B., Bekmanova G., Sharipbay A. (2017). Sentiment analysis on the hotel reviews in the Kazakh language. Proceedings of the 2017 International Conference on Computer Science and Engineering (UBMK).

[42] Yergesh B., Bekmanova G., Sharipbay A., Yergesh M. (2017). Ontology-based sentiment analysis of kazakh sentences. Proceedings of the Computational Science and Its Applications–ICCSA 2017: 17th International Conference.

[43] Bekmanova G., Yergesh B., Ukenova A., Omarbekova A., Mukanova A., Ongarbayev Y. Sentiment Processing of Socio-political Discourse and Public Speeches. Proceedings of the International Conference on Computational Science and Its Applications.

[44] Bekmanova G., Sairanbekova A., Ongarbayev Y., Mukanova A., Zulkhazhav A., Omarbekova A., Ukenova A. Intelligent question-answering system based on the public political discourse knowledge. Proceedings of the 2024 10th International Conference on e-Society, e-Learning and e-Technologies.

[45] Zhetkenbay L., Sharipbay A., Bekmanova G., Kamanur U. (2016). Ontological modeling of morphological rules for the adjectives in Kazakh and Turkish languages. J. Theor. Appl. Inf. Technol..

[46] Bekmanova G., Sharipbay A., Altenbek G., Adali E., Zhetkenbay L., Kamanur U., Zulkhazhav A. (2017). A Uniform Morphological Analyzer for the Kazakh and Turkish Languages. AIST (Supplement).

[47] Bekmanova G., Ukenova A., Omarbekova A., Zakirova A., Kantureyeva M. (2024). Features of the Interface of System for Solving Social Problems. Proceedings of the 2024 8th International Conference on Computer, Software and Modeling (ICCSM).

[48] Yergesh B., Kabdylova D., Mukhamet T. (2024). Semantic Knowledge Base for the Emotional Coloring Analysis of Kazakh Texts. Proceedings of the 2024 9th International Conference on Computer Science and Engineering (UBMK).

[49] Liang Y., Jettanasen C., Chiradeja P. (2024). Progression Learning Convolution Neural Model-Based Sign Language Recognition Using Wearable Glove Devices. Computation.

[50] Kamiya M., Guo Z. (2024). Scope of Negation, Gestures, and Prosody: The English Negative Quantifier as a Case in Point. J. Psycholinguist. Res..

[51] Mussakhojayeva S., Khassanov Y., Varol H.A. (2022). KazakhTTS2: Extending the open-source Kazakh TTS corpus with more data, speakers, and topics. arXiv.

[52] Abdo I.M.K.B. (2020). Computer Programming and Readability Scoring Tests between Arabic and English of Surat Al-Fātiḥ. J. Soc. Sci..

[53] Zhou S., Jeong H., Green P.A. (2017). How consistent are the best-known readability equations in estimating the readability of design standards?. IEEE Trans. Prof. Commun..

[54] Crossley S., Skalicky S., Berger C., Heidari A. (2022). Assessing readability formulas in the wild. Proceedings of the 7th Conference on Smart Learning Ecosystems and Regional Development.

[55] Nahatame S. (2021). Text readability and processing effort in second language reading: A computational and eye-tracking investigation. Lang. Learn..

[56] Relaño-Iborra H., Dau T. (2022). Speech intelligibility prediction based on modulation frequency-selective processing. Hear. Res..

[57] Ziemer T., Brian C.J. (2020). Psychoacoustics. Springer Handbook of Acoustics.

[58] Koffi E. (2023). A Comprehensive Review of Intonation: Psychoacoustics Modeling of Prosodic Prominence. Linguist. Portf..

[59] Prajwal K.R., Mukhopadhyay R., Namboodiri V.P., Jawahar C.V. A lip sync expert is all you need for speech to lip generation in the wild. Proceedings of the 28th ACM International Conference on Multimedia.

[60] Kalaian S.A., Kasim R.M. (2014). A meta-analytic review of studies of the effectiveness of small-group learning methods on statistics achievement. J. Stat. Educ..

[61] Quirk T.J. (2012). One-way analysis of variance (ANOVA). Excel 2007 for Educational and Psychological Statistics.

[62] Ganyaupfu E.M. (2013). Teaching methods and students’ academic performance. Int. J. Humanit. Soc. Sci. Invent..

[63] Gignac G.E., Szodorai E.T. (2016). Effect size guidelines for individual differences researchers. Personal. Individ. Differ..

[64] Joseph J. (2023). Assessing the potential of laboratory instructional tool through Synthesia AI: A case study on student learning outcome. Int. J. e-Learn. High. Educ..

[65] Mandal S., Ghosh B., Chakraborty S., Naskar R. (2024). Can Deepfakes Mimic Human Emotions? A Perspective on Synthesia Videos. Proceedings of the TENCON 2024—2024 IEEE Region 10 Conference (TENCON).

[66] Maria H.T., Aunurrahman A., Salam U., Karolina V., Warneri W., Nuraini D., Effendy S., Firmansyah T., Liana L., Pratiwi F. (2025). Integration of Artificial Intelligence with Studio D-ID for Development of Interactive Learning Media. I-Com Indones. Community J..

